# Spontaneous Belief Attribution in Younger Siblings of Children on the Autism Spectrum

**DOI:** 10.1037/a0034146

**Published:** 2013-08-26

**Authors:** Teodora Gliga, Atsushi Senju, Michèle Pettinato, Tony Charman, Mark H. Johnson, The BASIS Team

**Affiliations:** 1 Centre for Brain and Cognitive Development, Birkbeck, University of London, London, England; 2 Centre for Brain and Cognitive Development, Birkbeck, University of London, and Department of Speech, Hearing and Phonetic Sciences, University College London, London, England; 3 Institute of Psychiatry, King’s College London, London, England; 4 Centre for Brain and Cognitive Development, Birkbeck, University of London

**Keywords:** autism, family risk, false belief, eye-tracking

## Abstract

The recent development in the measurements of spontaneous mental state understanding, employing eye-movements instead of verbal responses, has opened new opportunities for understanding the developmental origin of “mind-reading” impairments frequently described in autism spectrum disorders (ASDs). Our main aim was to characterize the relationship between mental state understanding and the broader autism phenotype, early in childhood. An eye-tracker was used to capture anticipatory looking as a measure of false beliefs attribution in 3-year-old children with a family history of autism (at-risk participants, *n* = 47) and controls (control participants, *n* = 39). Unlike controls, the at-risk group, independent of their clinical outcome (ASD, broader autism phenotype or typically developing), performed at chance. Performance was not related to children’s verbal or general IQ, nor was it explained by children “missing out” on crucial information, as shown by an analysis of visual scanning during the task. We conclude that difficulties with using mental state understanding for action prediction may be an endophenotype of autism spectrum disorders.

It has been proposed that the social and communication impairments that characterize autism spectrum disorders (ASDs) stem from delays and/or difficulties with understanding that human actions are a result of mental states (e.g., desires, knowledge and beliefs), which may not always conform to reality (Baron-Cohen, 2005, but see Gernsbacher & Frymiare, 2005). Although “theory of mind” studies often show participants with ASD at a disadvantage, compared to control participants, exceptions do exist, which make it difficult to describe mindreading difficulties as a *core* deficit in ASD. For example, tasks that assess false belief understanding (e.g., the Sally-Anne task, the Smarties task) have repeatedly found that children with ASD fail at an age where typically developing children had long overcome any difficulties (Baron-Cohen, Leslie, & Frith, 1985; Leslie & Thaiss, 1992) but also that performance is dependent on linguistic abilities (Milligan, Astingdon, & Dack, 2007). Older children with ASD and/or those with more advanced language easily pass these tests (Bowler, 1992; Happé, 1995; Steele, Joseph, & Tager-Flusberg, 2003). This and the fact that difficulties with false belief understanding can also result from language difficulties, such as specific language impairment (SLI; Farrar et al., 2009; van Buijsen, Hendriks, Ketelaars, & Verhoeven, 2011), suggested that reasoning about mental states may be difficult only for less linguistically able individuals and is therefore neither specific nor universal for ASD.

However, a nonlinguistic false belief task, designed to test this ability in pre-verbal children (Southgate, Senju, & Csibra, 2007) has recently revealed difficulties with using mental states for action prediction, even in verbally able adults with autism (Senju, Southgate, White, & Frith, 2009). In this task an eye-tracker is used to monitor gaze behavior while participants watch a video clip depicting a false belief attribution scenario similar in structure to the Sally-Anne task. Instead of asking participants to verbally report where someone would look for an object displaced without that person knowing, eye-tracking is used to measure whether the participant looks in anticipation toward the location where the person is expected to search. Reality biases in responses (i.e., not being able to inhibit looking to the true location of the object instead of the location where the person would search) were prevented by having the object removed from the scene instead of just changing its location. These modifications allowed children as young as 2 years of age to succeed in this task and thus show evidence for mental state understanding (Southgate et al., 2007). In contrast to the good performance of typically developing toddlers, adults with Asperger syndrome and older children with autism perform at chance (Senju et al., 2009). Measures of anticipatory looking reflect spontaneous, on-line computation of others’ mental states, which may be distinct from and developing in parallel with the ability to reason about behaviors in terms of mental states when explicitly asked to do so. Predicting other people’s actions based on their mental states is as important for functioning in a social world as being able to reason and communicate about these mental states. Taking part in joint activities is just one example in which action prediction is believed to be instrumental (Sebanz & Knoblich, 2009). Unlike performance in classical Sally-Ann tasks, anticipatory looking is unrelated to language skills (Ruffman, Garnham, & Rideout, 2001; Senju et al., 2010) and is possibly present in typically developing infants as young as 7 months (Kovács, Teglas, & Endress, 2010).

The reliance on language skills to succeed in the standard theory of mind task has clouded our understanding of the relationship between mindreading difficulties and symptomatology of autism in terms of social and communication abilities. One strategy employed to investigate this relationship is to take advantage of the wider range of social abilities manifested by relatives of individuals with ASD. For example, Losh and Piven (2007) showed poor sociability in relatives was reflected in poor performance on the “Reading the Mind in the Eyes Test,” a test that requires inferring emotional mental states from only the eyes region of faces (Baron-Cohen & Hammer, 1997; Dorris, Espie, Knott, & Salt, 2004). In contrast, nonaffected siblings of children with autism performed as well as controls, in standard false belief tasks (Shaked, Gamliel, & Yirmiya, 2006). In this study, however, good performance could be explained by the good verbal and nonverbal skills siblings had, which may have allowed them to infer mental states off-line, when asked explicitly. However, we know that even those individuals with ASD capable of reasoning about a false belief situation like Sally-Anne may still have difficulties with using this information in real time (Senju et al., 2009).

It is the above considerations that motivated the current study in which action anticipation based on mental states understanding was measured in 3-year-old siblings of children with ASD, using the same paradigm as in Senju et al. (2009). Younger siblings manifest a wide variety of clinical and subclinical ASD-like traits. Recurrence rates in these populations vary, but in the largest study to date (*N* = 664 at-risk siblings) around 20% of at-risk participants developed ASD when assessed around 3 years of age (Ozonoff et al., 2011). Moreover, other siblings at high familial risk for autism, despite not reaching the clinical threshold for ASD, can subsequently manifest a wide range of social and communicative difficulties—considered to be manifestations of the broader autism phenotype (Rogers, 2009; Yirmiya & Charman, 2010). Showing difficulties in this task not only in those children with ASD but also in those on the broader autism phenotype will reinforce the hypothesis of a common origin for the social skills and the “mindreading” difficulties characteristic of ASD (Baron-Cohen et al., 1985).

Failure in mental state understanding tasks must be interpreted cautiously as difficulties with aspects of the tasks other than mental state understanding can result in poor performance. A lack of a bias or motivation to attend to socially relevant information as well as attention disengagement difficulties have both been proposed as alternative explanations for the apparent “mind-blindness” of people with autism. It has been suggested that people with ASD are not motivated to infer other people’s mental states and intentions (Andari et al., 2010; Dawson et al., 2004; Liebal, Colombi, Rogers, Warneken, & Tomasello, 2008), which could make them “miss out on” information needed to succeed in theory of mind tasks. Equally, failure could result from difficulties with disengaging from irrelevant aspects of the scene (Elsabbagh et al., 2009; Landry & Bryson, 2004) in order to notice when the actor attends or does not attend to the displacement of the object. Toddlers who eventually received a diagnosis of autism were also shown to explore objects in atypical ways, by placing them in their peripheral vision (Ozonoff et al., 2008). Because we used an eye-tracker to monitor gaze we were able to quantify differences in looking behavior during the task and thus address the above concerns. Senju et al. (2009) did not find a relationship between performance and gaze distribution in older children with ASD. Other studies on this population have found a dissociation between looking at and processing the looked-at information. For example although children at risk for ASD had no difficulties following someone’s gaze, they did not succeed in learning the name of the gazed-at object (Gliga, Elsabbagh, Hudry, Charman, & Johnson, 2012). We therefore expect no relationship between visual attention distribution and performance in the false belief task. As in the above-mentioned word learning study we expect all children, including poor performers, to attend to key events (e.g., to look at the actress when she turns away and cannot see an object being moved).

This study aims to characterize the relationship between mental state understanding and clinical and subclinical ASD profiles, in 3-year-olds with a family history of this disorder. For the first time with this population, we use eye-gaze as a measure of using mental state understanding for action prediction, a measure not confounded by children’s poor verbal skills. The nature of the clinical outcomes within the at-risk group offers the unique opportunity to test whether difficulties with mental state understanding are characteristic of children with a diagnosis of ASD only or of children with poor social and communicative abilities in general (the BAP). Having access to detailed gaze behavior during the task, a further aim of this study is to show that poor performance is not due to poor or atypical visual attention.

## Method

### Participants

Participants took part in a longitudinal study of children at risk for autism. Recruitment, ethical approval (London Research Ethics Committee, ref no. 09/H0718/14) and informed consent, as well as background data on participating families, were made available for the current study through BASIS, a UK collaborative network facilitating research with infants at risk for autism. Families enroll when their babies are younger than 5 months of age, and they are invited to attend multiple research visits until their children reach 3 years of age or beyond. Measures collected are anonymized and shared among scientists to maximize collaborative value and to minimize burden on the families. A clinical advisory team of senior consultants works closely together with the research team/s and, if necessary, with the families’ local health services, to ensure that any concerns about the child, arising during the study are adequately addressed. At the time of enrollment, none of the infants had been diagnosed with any medical or developmental condition. Of the initial 50 Control and 54 At-risk participants, 39 Control and 47 At-risk contributed data to this study. Two Control and one At-risk did not take part in the 36-months visit. Nine other Control were excluded because data were not collected due to technical problems (four) or because of having accumulated less than 20% looking data (five). Six At-risk participants were not included because of home visits (two), having accumulated less than 20% looking data (three) or being more than 1 year older than the group average at this visit (one). Participants’ characteristics (age, gender, IQ) are presented in Table 1.[Table-anchor tbl1]

At-risk infants had an older sibling (hereafter, proband) with a community clinical diagnosis of ASD (in three cases, a half-sibling), and in one case two probands with an ASD. Thirty-eight probands were male, nine were female. Proband diagnosis was confirmed by two expert clinicians (PB, TC) based on information using the Development and Well Being Assessment (DAWBA; Goodman, Ford, & Richards, 2000) and the parent-report Social Communication Questionnaire (SCQ; Rutter, Bailey, & Lord, 2003). The DAWBA is a parent-completed Web-based assessment that asks parents to rate symptoms of autism, relevant to making *Diagnostic and Statistical Manual of Mental Disorders* (4th ed., text rev.; *DSM–IV–TR*; American Psychiatric Association, 2000) and ICD-10 (World Health Organization, 1993) diagnosis of autism spectrum disorders. Descriptive information about the child is also included. The experts review the forms using both the scores and the narrative text to assign a diagnosis. The SCQ is a widely used 40-item questionnaire that asks about current and past autism symptoms. Most probands met criteria for ASD on both the DAWBA and SCQ (*n* = 42). While a small number scored below threshold on the SCQ (*n* = 4), no exclusions were made, due to meeting threshold on the DAWBA and expert opinion. For one proband, data were only available for the DAWBA. Parent-reported family medical histories were examined for significant medical conditions in the proband or extended family members, with no exclusions made on this basis. Infants in the Control group were recruited from a volunteer database. Inclusion criteria included full-term birth, normal birth weight, and lack of any ASD within first-degree family members (as confirmed through parent interview regarding family medical history). All Control infants had at least one older sibling (in three cases, only half-sibling/s). Screening for possible ASD in these older siblings was undertaken using the SCQ, with no child scoring above instrument cutoff for ASD (≥15). Sixty-two percent of controls and 85% of at-risk participants were only exposed to one language.

### Stimuli

**Figure 1 fig1:**
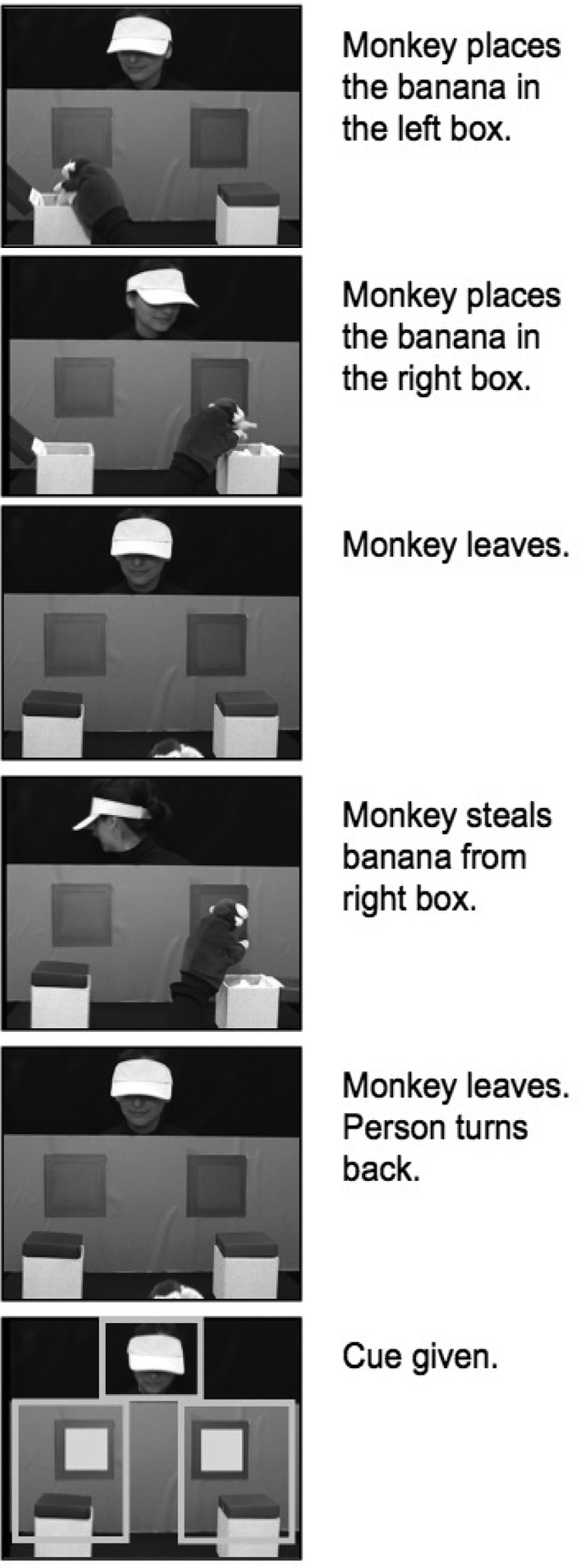
Key events during the false belief trial. The last frame depicts the three areas of interest used for data extraction.

The stimulus was a video recording, which depicted five main events: two familiarization trials, two true belief trials (TB) and one final false belief (FB) trial (see Figure 1). We familiarized children with two events in which an actress reached through two doors for a toy strawberry placed on the left (first trial) or the right of two boxes (second trial). An audiovisual cue (the windows were illuminated and a chime sounded) was given and 2.5 s later the actor reached through the window and grasped the strawberry. The actor wore a visor so that her gaze direction could not betray the direction of her reach through the windows. The purpose of the familiarization trials was (a) to show the children that the actor’s goal was to reach for the object and (b) to teach the children that when the audiovisual cue was presented one of the windows was about to open. At the beginning of the two TB trials, a puppet monkey appeared and placed a banana in the left box (first TB trial) or the right box (second TB trial). After leaving the scene and 2.5 s after the cue appeared the actress reached through the door behind the box that contained the banana. The FB trial is depicted in Figure 1. Crucially, in this trial, the actor turned away from the scene and the puppet monkey returned to remove the banana from the right side box, which induced a false belief in the actor. After the cue was given in this trial the scene froze for another 5 s. Because we could not counterbalance the locations of the banana in the FB trial within each outcome group (the outcome was not known at the time when the study was carried out), the same video clip was used for all participants.[Fig-anchor fig1]

### Procedure

An integrated Tobii (Stockholm, Sweden) T120 17” Eye Tracker was used to collect data on direction of gaze. Data were collected at 60 Hz. Tobii Studio was used to present the stimuli and for data analysis. Children sat on their own on a chair, at approximately 60 cm from the Tobii monitor. At this distance the diagonal of the screen subtended approximately 40°. A 5-point calibration was run before stimulus presentations. Children were told that they would see a movie about a cheeky monkey. An experimenter stood behind the child and encouraged her to look if she got distracted.

### Data Reduction and Analysis

The 2 min 45 s long video was segmented into scenes of various lengths corresponding to the various important events. To measure anticipatory eye-movements in the second true belief trial we defined a 2.5-s interval after the visual cue appeared, which corresponded roughly to the time taken for the person to reach through the doors in both the familiarization trials and the TB trials. Because the actor never reached through the door in the false belief trial, a 5-s interval (until the end of the movie) was used for analysis. For clarity, details about the length of other intervals analyzed are given together with the results of those particular analyses, in the Results section. Three areas of interest (AOI) were defined manually for all scenes analyzed (see Figure 1), two covering the left and right doors and boxes and another one corresponding to the face. Cumulative looking time within areas of interest was calculated automatically using Tobii Studio software. Only fixations longer than 100 ms were included in the analyses. Data loss could occur during the video presentation at different time points (either due to looking away or to the eye-tracker not detecting the eyes despite the fact that the child was looking). We decided to only exclude children if they accumulated less than 20% data overall and not if only certain intervals had valid data, the consequence of which was that slightly different numbers of participants were entered in the analysis of different events (e.g., in the TB and the FB trial analysis).

### Outcome Characterization of the At-Risk and Control Groups

Standard measures of cognitive development (Mullen Scales for Early Learning [MSEL]; Mullen, 1995) and adaptive development (Vineland Adaptive Behavior Scale [VABS]; Sparrow, Cicchetti, & Balla, 2005) were collected. The MSEL is a standardized direct developmental assessment that yields a standardized score (*M* = 100, *SD* = 15) of overall intellectual ability (Early Learning Composite, and subscale T-scores (*M* = 50, *SD* = 10) for receptive language (RL) and expressive language (EL), as well as nonverbal fine motor (FM) and visual reasoning (VR) abilities. The VABS is a standardized parent-reported interview of everyday adaptive functioning that measures social, communication, daily living and motor skills. In addition (and for both groups) a semistructured play-based assessment, the Autism Diagnostic Observation Schedule—Generic (ADOS-G; Lord et al., 2000) was used to assess autism-related social and communication behavioral characteristics (44 children were administered Module 2 and the other three children Module 1 of the ADOS-G). This was augmented (At-risk group only) with the parent-report Autism Diagnostic Interview—Revised (ADI-R; Lord et al., 1994). In common with other research groups studying familial at-risk siblings (Zwaigenbaum et al., 2007) a “best estimate clinical consensus” approach to diagnosis was taken following review by experienced clinical researchers (TC, KH, SC, GP), taking account of all information about the child (i.e., MSEL, VABS, informal observation) in addition to information from the ADI-R and ADOS-G. Children were included in the At-risk ASD group if they met ICD-10 (World Health Organization, 1993) criteria for ASD. Given the young age of the children, and in line with the proposed changes to the *Diagnostic and Statistical Manual of Mental Disorders* (5th ed.; *DSM-5*; http://www.dsm5.org), no attempt was made to assign specific subcategories of pervasive developmental disorder/ASD diagnosis. Children from the At-risk group were considered typically developing (At-risk Typical) if they (a) did not meet ICD-10 criteria for an ASD, (b) did not score above the ASD cutoff on the ADOS or ADI, (c) scored within 1.5 *SD* of the population mean on the Mullen Early Learning Composite (ELC) score (>77.5) and Receptive Language (RL) and Expressive Language (EL) subscale T scores (>35). Children from the At-risk group were considered to have atypical development if they did not fall into either of the above groups. That is, they either scored above the ADOS or ADI cutoff for ASD or scored < 1.5SD on the Mullen ELC or RL and EL but did not meet ICD-10 criteria for an ASD. From the 47 At-risk participants taking part in this task, 17 met criteria for an ASD diagnosis, 18 were At-risk Typical, and 12 were in the At-risk Atypical group (nine scoring above ADOS ASD cutoff, one scoring above ADOS ASD cutoff *and* <1 *SD* Mullen ELC cutoff, one scoring above ADI ASD cutoff, and one scoring <1.5 *SD* Mullen ELC cutoff).

## Results

We analyzed separately the true belief (TB) and the false belief (FB) trials. As in previous studies (Southgate et al., 2007), only the second TB trial was analyzed. By not including the first TB trial, we thus gave children more opportunities to understand the actor’s goal—to reach for the objects—as well as the role of the audiovisual cue. For each trial we assess performance by analyzing the difference between the looking time (LT) to the correct and incorrect doors’ AOIs, scaled by the amount of looking to those AOIs: (LT_Correct_ − LT_Incorrect_)/(LT_Correct_ + LT_Incorrect_). Values go from −1 (exclusive looking toward the Incorrect location) to 1 (exclusive looking to the Correct location), with chance level at zero. In the TB trial, the correct location was that which contained the banana. In the FB trial, the correct location was that in which the actor thought the banana was. We start the analysis by comparing the Control and At-risk groups to chance levels and to each other and then compare the three outcome groups within the At-risk participants (At-risk ASD, At-risk Atypical, and At-risk TD) to chance levels and to each other (using Bonferroni correction for multiple comparisons). Where a significant difference between Controls and At-risk is found, we also test whether all at-risk groups are significantly different than Controls (using Dunnett correction for multiple comparisons). We also test whether any individual variables that showed groups differences, like total IQ, verbal IQ, or age (see Table 1) explain group differences in TB or FB performance. Finally, we examine whether visual attention distribution during the false belief trial may account for children’s performance. Three AOIs were entered in this analysis, the door AOIs and another AOI corresponding to the actor (Figure 1).

### True Belief

Looking time differential scores were significantly above chance (zero) for both Control and At-risk participants: Control *t*(37) = 3.01, *p* = .005; At-risk *t*(40) = 2.99, *p* = .005. A univariate analysis of variance (ANOVA) with Group (At-risk vs. Control) as between-subjects factor yielded a nonsignificant effect of Group, *F*(1, 78) = 0.13, *p* = .716, η^2^ = .002. Verbal and General IQ significantly predicted performance—Verbal IQ *F*(1, 76) = 1.78, *p* = .03, η^2^ = .06; General IQ *F*(1, 76) = 1.78, *p* = .02, η^2^ = .06—however, entering these factors in the above ANOVA did not change the significance level for the main Group factor. When analyzing the behavior of the three at-risk groups separately, they only performed marginally better than chance—At-risk Typical *t*(15) = 1.24, *p* = .23; At-risk Atypical *t*(10) = 2.01, *p* = .07; At-risk ASD *t*(13) = 2.03, *p* = .06)—possibly also because of the reduced power of this analysis. A univariate ANOVA comparing the three at-risk subgroups (At-risk ASD, At-risk Atypical and At-risk Typical) yielded no significant effect of Group, *F*(2, 40) = .19, *p* = .82, η^*2*^ = .01.

### False Belief

**Figure 2 fig2:**
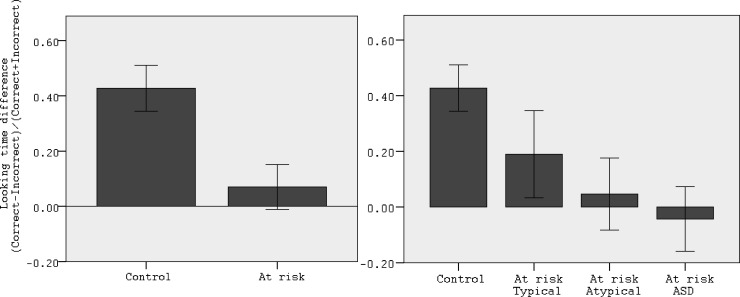
Looking time differential scores in the false belief trial. The chance level is at zero. ASD = autism spectrum disorder. Error bars represent standard error.

At the point at which anticipatory looking is measured in the False Belief trial the two boxes were empty, thus preventing a reality bias. Correct anticipation is reflected in longer looking toward the box that contained the banana just before the person looked away. As seen in Figure 2, looking time differential scores were higher for the Control group than for the High-risk groups. Preliminary analyses confirmed that Total IQ, Verbal IQ, or Age did not have a main effect on looking time distribution nor did they interact with the factor Group. We therefore removed these factors from further analyses. There was also no group difference in the overall amount of time spent looking at the three target AOIs (correct, incorrect, and face) after the light prompt, in the FB trial (*M*_*Control*_ = 3.35, *SD*_*Control*_ = 1.4 s; *M*_*At-risk*_ = 3.56, *SD*_*At-risk*_ = 1.15 s), *t*(82) = −0.71, *p* = .47. Mean looking time difference scores were significantly above chance only for the Control participants: Control *t*(35) = 5.13, *p* < .001; At-risk *t*(42) = 0.86, *p* = .39. A univariate ANOVA with Group (Control and At-risk) as between-subjects variable resulted in a significant main effect of Group, *F*(1, 78) = 9.35, *p* = .003, η^*2*^ = .10. The significance level of the Group factor did not change when the TB looking time performance was entered as a covariate and TB performance did not have a significant impact on FB performance (see Table 2). When the three At-risk subgroups (At-risk ASD, At-risk Atypical, and At-risk Typical) performance was analyzed separately, none of the groups performed different than chance: At-risk Typical *t*(16) = 1.21, *p* = .23; At-risk Atypical *t*(9) = 0.36, *p* = .72; At-risk ASD *t*(15) = –0.36, *p* = .71. A univariate ANOVA comparing the looking time difference scores for the three At-risk subgroups yielded a nonsignificant effect of Group, *F*(2, 42) = 0.79, *p* = .46, η^*2*^ = 0.03. Post hoc *t* tests, were used to compare each at-risk group to the Control participants. Only At-risk ASD significantly differed from Control participants (*p* = .009), At-risk Atypical and At-risk Typical were not significantly different from Control (*p* = .11 and *p* = .33).[Fig-anchor fig2][Table-anchor tbl2]

### Relationship With Social and Communication Abilities (ADOS)

The lack of a difference in performance between the three at-risk groups suggests that difficulties with mental state understanding may be unrelated to ASD symptom severity. To confirm that performance in this task is only related to the risk status and not to children’s social and communication abilities as measured by the ADOS, we split the Control and At-risk groups depending on their ADOS scores into a *Low ADOS* (ADOS < 8; 25 out of 35 Controls and 21 out of 42 At-risk participants) and *High ADOS* group (ADOS ≥ 8). Looking time performance was entered in a univariate ANOVA with Group (Control, At-risk) and ADOS (Low, High ADOS). This analysis yielded a main effect of risk Group, *F*(1, 76) = 9.41, *p* = .003, η^*2*^ = 0.11. The ADOS scores did not significantly predict performance, *F*(1, 76) = .19, *p* = .66, η^*2*^ = 0.003, and there was no significant interaction between risk Group and ADOS levels, *F*(1, 76) = 1.52, *p* = .22, η^*2*^ = 0.02.

### Differences in Attention to the Placement/Displacement Events

**Figure 3 fig3:**
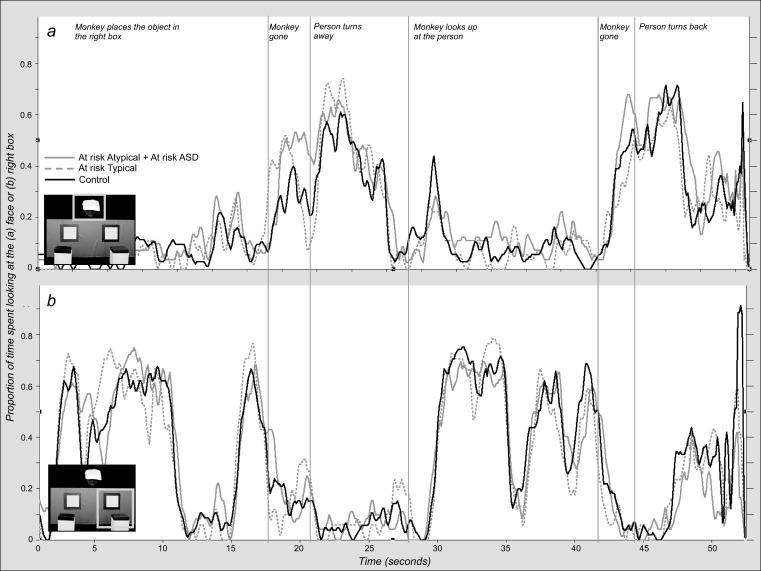
a. Proportion of children looking at the face area of interest (AOI) during the false belief (FB) trial. b. Proportion of children looking at the correct AOI (the right box) during the FB trial to illustrate all groups keeping track of where the banana was placed. At-risk autism spectrum disorder (ASD) and At-risk Atypical data were pooled together for clarity (continuous gray line). At-risk Typical (dashed gray line) and Controls (black line). The onset of important events is indicated on the time line.

What can explain the poorer performance of the At-risk participants in the FB trial? We were interested in determining whether children’s looking behavior during the task differed in any way that would explain their performance. One possible source of error could arise from not paying attention to the hiding and displacement events during the FB interval, especially the last hiding event before the actress looks away. Visual inspection of looking time distribution along the FB trial suggests that all groups followed closely this event (Figure 3a: “Banana placed in the right box”; “Monkey steals banana from right box”) and that major differences between groups only emerge at the very end, when FB is tested (Figure 3a: “Person turns back”). We looked more specifically at attention distribution during key events. Groups spent equal amounts of time looking at the box during the 8 s that the monkey took to place the banana (*M*_*Control*_ = 4.9, *SD*_*Control*_ = 2.0; *M*_*At-risk Typical*_ = 5.3, *SD*_*At-risk Typical*_ = 1.6; *M*_*At-risk Atypical*_ = 4.9, *SD*_*At-risk Atypical*_ = 1.7; *M*_*At-risk ASD*_ = 5.3, *SD*_*At-risk ASD*_), *F*(3, 84) = 0.51, *p* = .67, η^*2*^ = .01. Groups also spent equal amounts of time looking at the box from which the monkey surreptitiously removed the banana (*M*_*Control*_ = 7.4, *SD*_*Control*_ = 2.9; *M*_*At-risk Typical*_ = 6.5, *SD*_*At-risk Typical*_ = 3.0; *M*_*At-risk Atypical*_ = 7.9, *SD*_*At-risk Atypical*_ = 2.3; *M*_*At-risk ASD*_ = 6.7, *SD*_*At-risk ASD*_ = 2.7), *F*(3, 84) = 0.91, *p* = .44, η^*2*^ = .03. It is also important to have noticed that, when the banana was removed from the box, the person was looking away. Visual inspection of looking time spent on the face during the FB trial does not highlight consistent group differences (Figure 3b), and, indeed, when we compared the amount of time spent looking at the person’s face while the monkey removed the banana from the box no group difference was found (*M*_*Control*_ = 3.8, *SD*_*Control*_ = 1.9; *M*_*At-risk Typical*_ = 3.1, *SD*_*At-risk Typical*_ = 2.4; *M*_*At-risk Atypical*_ = 4.0, *SD*_*At-risk Atypical*_ = 2.7; *M*_*At-risk ASD*_ = 4.5, *SD*_*At-risk ASD*_ = 2.6), *F*(3, 84) = 1, *p* = .41, η^*2*^ = .03. None of these measures correlate with the FB looking time difference score, for either the whole group or the low and at-risk groups separately.[Fig-anchor fig3]

Closer exploration of the data revealed that at the point in the video where the monkey had placed the banana in the right box and left the screen and before the person turned away, children looked up at the person (*M*_*Control*_ = 2.0, *SD*_*Control*_ = 1.1; *M*_*At-risk Typical*_ = 2.2, *SD*_*At-risk Typical*_ = 1.0; *M*_*At-risk Atypical*_ = 2.7, *SD*_*At-risk Atypical*_ = 1.3; *M*_*At-risk ASD*_ = 2.1, *SD*_*At-risk ASD*_ = 0,9), *F*(3, 80) = 1.42, *p* = .24, η^*2*^ = .05, and then looked toward the right door and box. Encoding where the person last saw the object or her goal at this point where a TB is still held may be crucial for predicting their behavior later. We analyzed looking time distribution to correct (here where the banana had been placed) and incorrect locations at this time point. Both Low-risk and High-risk participants looked longer at the Correct side—average and *SD* for Correct versus Incorrect for Low-risk: 620 ms (105) versus 370 ms (81) and High-risk: 552 ms (90) versus 369 ms (69). A 2 × 2 ANOVA with Side and Group confirmed that there was a main effect of Side, *F*(1, 79) = 5.01, *p* = .02, η^2^ = .06, but no main effect of Group, *F*(1, 79) = 0.21, *p* = .64, and no interaction between Side and Group, *F*(1, 79) = 0.11, *p* = .74, which means that both groups looked longer at the box containing the banana. To investigate whether looking time distribution at this point was related to performance later in the FB trial we calculated difference scores in both cases (Looking time Correct − Looking time Incorrect). These measures were correlated in the whole sample, *r*(70) = .27, *p* = .01, as well as in the Low-risk group, *r*(31) = .40, *p* = .02, but not in the High-risk group, *r*(39) = .13, *p* = .41. A Chow test demonstrated that the slope and intercept of the regression analysis predicting test performance from looking time distribution when the person last saw the object was not significantly different for the high-risk and low-risk participants, *F*(1, 69) = 2.02, *p* = .15.

## Discussion

Previous studies of mental state understanding have documented difficulties with on-line computation of mental states in older children with ASD (Senju et al., 2010) as well as in adults with ASD (Senju et al., 2009). Here we provide evidence that this impairment is measurable as early as 3 years of age in children at familial risk for this disorder and that it is not restricted to those children having received a diagnosis of ASD. Control participants, as a group, performed above chance, confirming previous findings at 24 months of age (Southgate et al., 2007) and suggesting this ability is continuously present during development from 2 years of age through adulthood (Senju et al., 2009, 2010). The At-risk participants, included in the study on the basis of having an older sibling with ASD, developed a wide range of social and communication abilities by 3 years of age, with some children receiving a diagnosis of ASD and others manifesting other developmental problems, including subclinical scores on the ADOS-G, which measures ASD-like social and communication atypicalities.

Based on previous findings of subtle difficulties with inferring mental states in relatives with poor sociability (Losh & Piven, 2007), we hypothesized that all children with poor social and communication abilities, i.e., both At-risk ASD and At-risk Atypical groups, would show difficulties with mental state understanding. Interestingly, all groups of at-risk children found the task difficult, including those at risk who developed typically. Moreover, performance was not related to social and communication abilities, as measured by the ADOS. The performance of the At-risk Typical group was not significantly different from that of the other at-risk groups but was also not different from that of Controls, suggesting that they may have intermediary abilities, with more participants succeeding at the task than in the other at-risk groups. Notwithstanding these findings, the At-risk Typical group’s performance was not significantly different than chance. Although similar in terms of IQ to Controls, the At-risk Typical group is more similar to the other at-risk participants in terms of both genetic and family background.

The picture of autism emerging from recent genetic studies is of a multifactorial disorder, in which outcomes are a result not of a small number of deterministic factors but of the combination of a great number of risk and protective factors (Geschwind, 2011). This model is supported by recent findings from prospective studies of infants at risk (Elsabbagh & Johnson, 2010). Difficulties with mental state understanding could be one of these many risk factors, which impacts on symptom severity only in combination with other concurrent factors. Family environment is expected to mediate shared genetic influences on the outcome phenotype both in terms of mental state understanding and social and communication abilities. Previous studies have shown that having an older sibling positively impacts on the development of mental state understanding in typically developing children (Ruffman, Perner, Naito, Parkin, & Clements, 1998). Many of our at-risk participants did not have a typically developing older sibling (they only had an older sibling with ASD). This may place them at a disadvantage in mindreading abilities with respect to Control participants, all of whom had a typically developing older sibling. At this point our sample is too small to properly investigate the interaction between genetic and environmental factors, something future studies will have to clarify.

Although we take failure in our task to mean difficulties with computing and using mental states, alternative explanations are possible. The design of the task, in particular the existence of a True Belief condition, allows us to rule out some of them. Above chance performance in the True Belief trial is evidence that both controls and at-risk participants understood the task and were motivated and able to anticipate someone’s actions. Although they did not look to the correct location, a majority of at-risk participants (43/47) did look toward one of the two possible locations in response to the audiovisual cue, in the False Belief trial, which again is not compatible with a lack of motivation. Using eye-tracking we could also ask whether performance can be explained by any differences in the looking distribution during the task. It has previously been proposed that attention disengagement difficulties or a lack of a bias to attend to social information can result in missing crucial information necessary to succeed in theory of mind tasks (Dawson et al., 2004). This would be even more problematic in on-line assessments of mental state understanding, than in classical, slower paced tasks. We therefore analyzed looking behavior at various points during the false belief trial and showed that all children looked at the object placement and displacement actions and also looked at the person when she turned away from the scene. There was thus no difference in the way children with ASD attended to the sequence of actions. More important, the amount of looking did not correlate with looking time performance at test, confirming our initial hypothesis that poor performance was not due to poor attention. Similarities in looking behavior in response to various key events (e.g., looking at the person when she turned away or at the monkey when she was engaged with the banana) also speak against any oculomotor differences between groups (Ozonoff et al., 2008). Of course, looking is necessary but not sufficient for attending to and processing the information fixated. As previously shown, in a word learning task, children at risk for ASD could follow someone’s gaze to an object but did not learn the word–object association as well as controls (Gliga et al., 2012). Brain imaging studies of face processing have also shown that even when asked to fixate faces, ASD participants activate a less extensive network of brain areas than neurotypical participants (Hadjikhani, Joseph, Snyder, & Tager-Flusberg, 2007). Thus, similarities in scanning of visual scenes in autism may mask learning and processing differences associated with this condition.

Some differences in looking behavior did appear during the false belief trial. When visually inspecting the data we noticed that children made saccades toward the location containing the banana earlier during the FB trial, before the cue was given, at the point where the person still held a true belief and could have reached for the box. At this point groups were again indistinguishable, suggesting that they had again correctly encoded the person’s reaching goal. The positive correlation between looking toward the box containing the banana at this point and looking toward this same box later in the trial, when the banana had been removed, suggests that, at least in controls, success in the FB trial depends on how well one encodes the goal of the actress at the moment at which it still corresponds to a true belief. This is compatible with one current model that explains spontaneous mental state attribution on the basis of corepresentations of people and the objects they encounter and act upon (Apperly & Butterfill, 2009; Samson, Apperly, Braithwaite, Andrews, & Bodley Scott, 2010). Thus, when someone repeatedly reaches for a particular object, their goals or beliefs about that object’s location are stored in memory together with that person’s identity for later reenactment. However, this correlation only holds in the control group. The lack of a correlation in the at-risk participants could reflect encoding of different information when children look at the box just before the person turns away (e.g., the location of the banana instead of the person’s goal) or difficulties with maintaining that information in memory. Future behavioral studies could test the impact of memory by varying the time delay between hiding, displacement and test. Brain imaging studies could measure the nature of the information encoded initially (e.g., object location or action goal). Although at this point we cannot tell whether these differences in performance are due to at-risk participants not being able to compute mental states nor whether they are due to not being able to keep in memory someone’s representation of the world while monitoring changes in the world itself, we subscribe to a recently made case for the importance of theory of mind tasks to reveal not just conceptual understanding but also functional usage of these abilities (Apperly, 2012). Whether they can compute mental states, we show that children with a family background of autism did not use these abilities online to anticipate another’s actions, which is of importance knowing that action prediction is believed to be crucial for joint activities (e.g., for example Sebanz & Knoblich, 2009).

The current study advances our understanding of mental state attribution in ASD by providing the earliest evidence that these difficulties are not restricted to those children that fulfill diagnostic criteria for ASD but characterize the whole at-risk group. It is unclear at this point whether these difficulties are specific to ASD risk, whether they reflect a genetic susceptibility, the influence of the social environment or maybe the interaction between these two factors. Difficulties with classical theory of mind tasks have been documented in other developmental disorders like Down syndrome or mental retardation (Yirmiya, Solomonica-Levi, Shulman, & Pilowsky, 1996). However, in these populations performance was correlated with nonverbal IQ, which suggests general cognitive factors like memory and attention can limit mental state understanding. This was not the case in our study, where general and verbal IQ did not explain group differences, nor did visual attention distribution during the task. A detailed analysis of looking behavior confirmed that failure was not due to “missing out” on important information, such as where the object had been placed/displaced or whether the person was attending to the scene or not. Therefore, success on this task does not appear to be due to where someone looks for information, but to how or whether the “looked at” information is later used.

The mechanisms of mental state understanding have been subject to heated debates both within autism research and within developmental psychology. We believe that both fields will benefit from the study of younger siblings of children with autism. The greater variability of social, communicative and attentional abilities this group manifests will make it possible to identify necessary conditions for the development and online use of mental state understanding. For example, while succeeding in standard false belief task depends on language skills, this is not the case for our task. Being able to encode and maintain in memory action goals while events unfold seems to be the limiting factor when mental states are used for action anticipation. Moreover, the wider variety of clinical outcomes in this population will help settle debates about the specificity of mindreading difficulties to autism spectrum disorders.

## Figures and Tables

**Table 1 tbl1:** Participants’ Characteristics

Variable	Control (*n* = 39)	At-risk (*n* = 47)	At-risk typical (*n* = 18)	At-risk atypical (*n* = 12)	At-risk ASD (*n* = 17)
Age (months)					
*M* (*SD*)	39.5 (3.3)	38.0 (1.7)*	37.9 (1.5)	37.5 (1.7)	38.6 (2.0)
Range	36–52	33–42	34–40	33–40	35–42
Gender					
Male	21	27	12	9	6
Female	18	20	6	3	11
General IQ (Mullen)^a^					
*M* (*SD*)	114.7 (16.1)	104.2 (22.5)*	113.2 (15.0)	103.4 (18.9)	94.7 (28.5)^++^
Range	72–137	49–147	86–142	63–126	49–147
Verbal IQ (Mullen)^b^					
*M* (*SD*)	57.5 (8.5)	51.2 (12.0)*	55.9 (14.5)	49.8 (8.6)	47.2 (15.9)
Range	41–69	29–73	43–69	29–64	20–73
ADOS SC^c^					
*M* (*SD*)	5.5 (4.5)	8.8 (5.3)**	4.1 (2.0)	12.0 (4.0)^++^	11.4 (5.0)^++^
Range	0–21	0–19	0–7	5–18	1–19
ADI Social					
*M* (*SD*)		5.0 (5.4)	1.9 (1.7)	3.4 (4.9)	9.7 (5.5)^++^
Range			0–5	0–18	1–19
ADI Communication					
*M* (*SD*)		4.5 (4.9)	2.2 (1.8)	3.5 (5.4)	8.2 (5.2)^++^
Range			0–6	0–20	1–16
Ethnicity					
Caucasian	33	41	17	9	14
Caucasian/Asian	2	2		1	1
Caucasian/Latino		1		1	1
Caucasian/Black	1	1			1
Asian	1				
Black	1	1		1	
Mixed	1	1	1		
Income^d^					
<40K	9	17	4	8	5
40–80K	14	23	8	3	12
>80K	16	7	6	1	0
Education^e^					
*M* (*SD*)	3.0 (0.9)	2.7 (0.9)	2.8 (0.9)	3.0 (0.9)	2.4 (0.9)
Range	1–4	1–4	1–4	1–4	1–4
*Note*. ASD = autism spectrum disorder; Mullen = Mullen Scales for Early Learning (Mullen, 1995); ADOS = Autism Diagnostic Observation Schedule—Generic (Lord et al., 2000); ADI = Autism Diagnostic Interview—Revised (Lord et al., 1994). Superscripts on data indicate differences between low and high risk (* *p* < .05; ** *p* < .01) and between the at-risk ASD or atypical groups and at-risk TD (^+^ *p* < .05; ^++^ *p* < .01; Bonferroni correction).					
^a^ Mullen ELC score, *M* = 100, *SD* = 15. ^b^ Verbal ability T-score, *M* = 50, *SD* = 10 (based on average of Expressive and Receptive Language domains). ^c^ The Social and Communication algorithm score of the ADOS. ^d^ Overall household income. ^e^ Mother’s education level (1 = *formal education to 16*; 2 = *formal education to 18*; 3 = *university degree or equivalent*; 4 = *postgraduate*). Missing data for Education level (seven data points) were replaced by the average of the risk group.					

**Table 2 tbl2:** Looking Time Differential Scores in the True Belief and False Belief Trials

Variable	Control	At-risk	At-risk typical	At-risk atypical	At-risk ASD
True belief					
*M*	.30*	.25*	.19	.32	.28
*SD*	.62	.55	.61	.52	.52
*N*	38	41	16	11	14
False belief					
*M*	.42*	.07	.18	.04	−.04
*SD*	.49	.53	.64	.40	.46
*N*	36	43	17	10	16
*Note*. ASD = autism spectrum disorder. The asterisk indicates significance of one-sample *t* tests (*p* < .05) against a chance level of zero.					

## References

[c53] American Psychiatric Association (2000). Diagnostic and statistical manual of mental disorders (4th ed., text rev.). Washington, DC: Author

[c1] Andari E., Duhamel J. R., Zalla T., Herbrecht E., Leboyer M., & Sirigu A. (2010). Promoting social behavior with oxytocin in high-functioning autism spectrum disorders. Proceedings of the National Academy of Sciences of the United States of America, 107, 4389–4394. doi:10.1073/pnas.0910249107 20160081 PMC2840168

[c2] Apperly I. A. (2012). What is “theory of mind”? Concepts, cognitive processes and individual differences. The Quarterly Journal of Experimental Psychology, 65, 825–839. doi:10.1080/17470218.2012.676055 22533318

[c3] Apperly I. A., & Butterfill S. A. (2009). Do humans have two systems to track beliefs and belief-like states? Psychological Review, 116, 953–970. doi:10.1037/a0016923 19839692

[c4] Baron-Cohen S. (2005). Theory of mind and autism: A fifteen year review In Baron-Cohen S., Tager-Flusberg H., & Cohen D. J. (Eds.), Understanding other minds: Perspectives from developmental cognitive neuroscience (pp. 3–20). New York, NY: Oxford University Press

[c5] Baron-Cohen S., & Hammer J. (1997). Parents of children with Asperger syndrome: What is the cognitive phenotype? Journal of Cognitive Neuroscience, 9, 548–554. doi:10.1162/jocn.1997.9.4.548 23968217

[c6] Baron-Cohen S., Leslie A. M., & Frith U. (1985). Does the autistic child have a “theory of mind”? Cognition, 21, 37–46. doi:10.1016/0010-0277(85)90022-8 2934210

[c7] Bowler D. M. (1992). Theory of mind in Asperger syndrome. Child Psychology & Psychiatry & Allied Disciplines, 33, 877–893. doi:10.1111/j.1469-7610.1992.tb01962.x 1378848

[c10] Dawson G., Toth K., Abbott R., Osterling J., Munson J., Estes A., & Liaw J. (2004). Early social attention impairments in autism: Social orienting, joint attention, and attention to distress. Developmental Psychology, 40, 271–283. doi:10.1037/0012-1649.40.2.271 14979766

[c11] Dorris L., Espie C. A. E., Knott F., & Salt J. (2004). Mind-reading difficulties in the siblings of people with Asperger’s syndrome: Evidence for a genetic influence in the abnormal development of a specific cognitive domain. Journal of Child Psychology and Psychiatry, 45, 412–418. doi:10.1111/j.1469-7610.2004.00232.x 14982254

[c12] Elsabbagh M., & Johnson M. (2010). Getting answers from babies about autism. Trends in Cognitive Sciences, 14, 81–87. doi:10.1016/j.tics.2009.12.005 20074996

[c13] Elsabbagh M., Volein A., Holmboe K., Tucker L., Csibra G., Baron-Cohen S., . . . Johnson M. H. (2009). Visual orienting in the early broader autism phenotype: Disengagement and facilitation. Journal of Child Psychology and Psychiatry, 50, 637–642. doi:10.1111/j.1469-7610.2008.02051.x 19298466 PMC3272379

[c14] Farrar M. J., Johnson B., Tompkins V., Easters M., Zilisi-Medus A., & Benigno J. P. (2009). Language and theory of mind in preschool children with specific language impairment. Journal of Communication Disorders, 42, 428–441. doi:10.1016/j.jcomdis.2009.07.001 19647837

[c16] Gernsbacher M. A., & Frymiare J. (2005). Does the autistic brain lack core modules? Journal of Developmental and Learning Disorders, 9, 3–16 PMC4266369 25520587

[c17] Geschwind D. H. (2011). Genetics of autism spectrum disorders. Trends in Cognitive Sciences, 15, 409–416. doi:10.1016/j.tics.2011.07.003 21855394 PMC3691066

[c18] Gliga T., Elsabbagh M., Hudry K., Charman T., & Johnson M. (2012). Gaze-following, gaze reading and word learning in children at-risk for autism. Child Development, 83, 926–938. doi:10.1111/j.1467-8624.2012.01750.x 22462503

[c19] Goodman R., Ford T., & Richards H. (2000). The Development and Wellbeing Assessment: Description and initial validation of an integrated assessment of child and adolescent psychopathology. Journal of Child Psychology and Psychiatry, 41, 645–656. doi:10.1111/j.1469-7610.2000.tb02345.x 10946756

[c20] Hadjikhani N., Joseph R. M., Snyder J., & Tager-Flusberg H. (2007). Abnormal activation of the social brain during face perception in autism. Human Brain Mapping, 28, 441–449. doi:10.1002/hbm.20283 17133386 PMC6871469

[c21] Happé F. G. (1995). The role of age and verbal ability in the theory of mind task performance of subjects with autism. Child Development, 66, 843–855. doi:10.2307/1131954 7789204

[c22] Kovács A. M., Teglas E., & Endress A. D. (2010). The social sense: Susceptibility to others’ beliefs in human infants and adults. Science, 330, 1830–1834. doi:10.1126/science.1190792 21205671

[c24] Landry R., & Bryson S. (2004). Impaired disengagement of attention in young children with autism. Journal of Child Psychology and Psychiatry, 45, 1115–1122. doi:10.1111/j.1469-7610.2004.00304.x 15257668

[c25] Leslie A. M., & Thaiss L. (1992). Domain specificity in conceptual development: Neuropsychological evidence from autism. Cognition, 43, 225–251. doi:10.1016/0010-0277(92)90013-8 1643814

[c26] Liebal K., Colombi C., Rogers S. J., Warneken F., & Tomasello M. (2008). Helping and cooperation in children with autism. Journal of Autism and Developmental Disorders, 38, 224–238. doi:10.1007/s10803-007-0381-5 17694374 PMC2758368

[c27] Lord C., Risi S., Lambrecht L., Cook E. H. Jr., Leventhal B. L., DiLavore P. C., . . . Rutter M. (2000). The autism diagnostic observation schedule-generic: A standard measure of social and communication deficits associated with the spectrum of autism. Journal of Autism and Developmental Disorders, 30, 205–223. doi:10.1023/A:1005592401947 11055457

[c54] Lord C., Rutter M., & Le Couteur A. (1994). Autism Diagnostic Interview–Revised: A revised version of a diagnostic interview for caregivers of individuals with possible pervasive developmental disorders. Journal of Autism and Developmental Disorders, 24, 659–685 7814313 10.1007/BF02172145

[c28] Losh M., & Piven J. (2007). Social-cognition and the broad autism phenotype: Identifying genetically meaningful phenotypes. Journal of Child Psychology and Psychiatry, 48, 105–112. doi:10.1111/j.1469-7610.2006.01594.x 17244276

[c30] Milligan K., Astingdon J. W., & Dack L. (2007). Language and theory of mind: Meta-analysis of the relation between language ability and false-belief understanding. Child Development, 78, 622–646. doi:10.1111/j.1467-8624.2007.01018.x 17381794

[c31] Mullen B. (1995). Mullen Scales of Early Learning. Circle Pines, MN: American Guidance Services

[c32] Mundy P., Sigman M., & Kasari C. (1990). A longitudinal study of joint attention and language development in autistic children. Journal of Autism and Developmental Disorders, 20, 115–128. doi:10.1007/BF02206861 2324051

[c34] Ozonoff S., Macari S., Young G. S., Goldring S., Thompson M., & Rogers S. J. (2008). Atypical object exploration at 12 months of age is associated with autism in a prospective sample. Autism, 12, 457–472. doi:10.1177/1362361308096402 18805942 PMC2921192

[c55] Ozonoff S., Young G. S., Carter A., Messinger D., Yirmiya N., Zwaigenbaum L., . . . Stone W. L. (2011). Recurrence risk for autism spectrum disorders: A Baby Siblings Research Consortium Study. Pediatrics, 128, e488–e495. doi:10.1542/peds.2010-2825 21844053 PMC3164092

[c35] Rogers S. J. (2009). What are infant siblings teaching us about autism in infancy? Autism Research, 2, 125–137. doi:10.1002/aur.81 19582867 PMC2791538

[c36] Ruffman T., Garnham W., & Rideout P. (2001). Social understanding in autism: Eye gaze as a measure of core insights. Journal of Child Psychology and Psychiatry, 42, 1083–1094. doi:10.1111/1469-7610.00807 11806690

[c37] Ruffman T., Perner J., Naito M., Parkin L., & Clements W. A. (1998). Older (but not younger) siblings facilitate false belief understanding. Developmental Psychology, 34, 161–174. doi:10.1037/0012-1649.34.1.161 9471013

[c38] Rutter M., Bailey A., & Lord C. (2003). Social Communication Questionnaire. Los Angeles, CA: Western Psychological Services

[c39] Samson D., Apperly I. A., Braithwaite J. J., Andrews B. J., & Bodley Scott S. E. (2010). Seeing it their way: Evidence for rapid and involuntary computation of what other people see. Journal of Experimental Psychology: Human Perception and Performance, 36, 1255–1266. doi:10.1037/a0018729 20731512

[c40] Sebanz N., & Knoblich G. (2009). Prediction in joint action: What, when and where. Topics in Cognitive Science, 1, 353–367. doi:10.1111/j.1756-8765.2009.01024.x 25164938

[c41] Senju A., Southgate V., Miura Y., Matsui T., Hasegawa T., Tojo Y., . . . Csibra G. (2010). Absence of spontaneous action anticipation by false belief attribution in children with autism spectrum disorder. Development and Psychopathology, 22, 353–360. doi:10.1017/S0954579410000106 20423546

[c42] Senju A., Southgate V., White S., & Frith U. (2009). Mindblind eyes: An absence of spontaneous theory of mind in Asperger syndrome. Science, 325, 883–885. doi:10.1126/science.1176170 19608858

[c43] Shaked M., Gamliel I., & Yirmiya N. (2006). Theory of mind abilities in young siblings of children with autism. Autism, 10, 173–187. doi:10.1177/1362361306062023 16613866

[c44] Skuse D. (2001). Endophenotypes and child psychiatry. The British Journal of Psychiatry, 178, 395–396. doi:10.1192/bjp.178.5.395 11331550

[c45] Southgate V., Senju A., & Csibra G. (2007). Action anticipation through attribution of false belief by 2-year-olds. Psychological Science, 18, 587–592. doi:10.1111/j.1467-9280.2007.01944.x 17614866

[c46] Sparrow S. S., Cicchetti D. V., & Balla D. A. (2005). Vineland Adaptive Behavior Scales: Survey Form (2nd ed.). Circle Pines, MN: American Guidance Service

[c47] Steele S., Joseph R. M., & Tager-Flusberg H. (2003). Brief report: Developmental change in theory of mind abilities in children with autism. Journal of Autism and Developmental Disorders, 33, 461–467. doi:10.1023/A:1025075115100 12959426

[c49] van Buijsen M., Hendriks A., Ketelaars M., & Verhoeven L. (2011). Assessment of theory of mind in children with communication disorders: Role of presentation mode. Research in Developmental Disabilities, 32, 1038–1045. doi:10.1016/j.ridd.2011.01.036 21349688

[c56] World Health Organization (1993). The ICD-10 classification of mental and behavioural disorders In Diagnostic criteria for research. Geneva, Switzerland: Author

[c50] Yirmiya N., & Charman T. (2010). The prodrome of autism: Early behavioral and biological signs, regression, peri- and post-natal development and genetics. Journal of Child Psychology and Psychiatry, 51, 432–458. doi:10.1111/j.1469-7610.2010.02214.x 20085609

[c51] Yirmiya N., Solomonica-Levi D., Shulman C., & Pilowsky T. (1996). Theory of mind abilities in individuals with autism, Down syndrome, and mental retardation of unknown etiology: The role of age and intelligence. Child Psychology & Psychiatry & Allied Disciplines, 37, 1003–1014. doi:10.1111/j.1469-7610.1996.tb01497.x 9119934

[c52] Zwaigenbaum L., Thurm A., Stone W., Baranek G., Bryson S., Iverson J., . . . Sigman M. (2007). Studying the emergence of autism spectrum disorders in high-risk infants: Methodological and practical issues. Journal of Autism and Developmental Disorders, 37, 466–480. doi:10.1007/s10803-006-0179-x 16897376

